# Development of Chitosan-Based Surfaces to Prevent Single- and Dual-Species Biofilms of *Staphylococcus aureus* and *Pseudomonas aeruginosa*

**DOI:** 10.3390/molecules26144378

**Published:** 2021-07-20

**Authors:** Marta Lima, Rita Teixeira-Santos, Luciana C. Gomes, Sara I. Faria, Jesus Valcarcel, José Antonio Vázquez, Miguel A. Cerqueira, Lorenzo Pastrana, Ana I. Bourbon, Filipe J. Mergulhão

**Affiliations:** 1 LEPABE-Laboratory for Process Engineering, Environment, Biotechnology and Energy, Faculty of Engineering, University of Porto, Rua Dr. Roberto Frias, 4200-465 Porto, Portugal; up201604683@fe.up.pt (M.L.); ritadtsantos@fe.up.pt (R.T.-S.); luciana.gomes@fe.up.pt (L.C.G.); sisf@fe.up.pt (S.I.F.); 2 Grupo de Reciclado y Valorización de Materiales Residuales (REVAL), Instituto de Investigaciones Marinas (IIM-CSIC), C/Eduardo Cabello, 6, CP36208 Vigo, Galicia, Spain; jvalcarcel@iim.csic.es (J.V.); jvazquez@iim.csic.es (J.A.V.); 3 International Iberian Nanotechnology Laboratory, Department of Life Sciences, Av. Mestre José Veiga s/n, 4715-330 Braga, Portugal; miguel.cerqueira@inl.int (M.A.C.); lorenzo.pastrana@inl.int (L.P.); ana.bourbon@inl.int (A.I.B.)

**Keywords:** antibiofilm activity, chitosan, chitosan-polylactic acid surfaces, dual-species biofilms, implantable medical devices, polylactic acid surfaces, *Pseudomonas aeruginosa*, single-species biofilms, *Staphylococcus aureus*

## Abstract

Implantable medical devices (IMDs) are susceptible to microbial adhesion and biofilm formation, which lead to several clinical complications, including the occurrence of implant-associated infections. Polylactic acid (PLA) and its composites are currently used for the construction of IMDs. In addition, chitosan (CS) is a natural polymer that has been widely used in the medical field due to its antimicrobial and antibiofilm properties, which can be dependent on molecular weight (Mw). The present study aims to evaluate the performance of CS-based surfaces of different Mw to inhibit bacterial biofilm formation. For this purpose, CS-based surfaces were produced by dip-coating and the presence of CS and its derivatives onto PLA films, as well surface homogeneity were confirmed by contact angle measurements, Fourier transform infrared spectroscopy (FTIR) and scanning electron microscopy (SEM). The antimicrobial activity of the functionalized surfaces was evaluated against single- and dual-species biofilms of *Staphylococcus aureus* and *Pseudomonas aeruginosa*. Chitosan-based surfaces were able to inhibit the development of single- and dual-species biofilms by reducing the number of total, viable, culturable, and viable but nonculturable cells up to 79%, 90%, 81%, and 96%, respectively, being their activity dependent on chitosan Mw. The effect of CS-based surfaces on the inhibition of biofilm formation was corroborated by biofilm structure analysis using confocal laser scanning microscopy (CLSM), which revealed a decrease in the biovolume and thickness of the biofilm formed on CS-based surfaces compared to PLA. Overall, these results support the potential of low Mw CS for coating polymeric devices such as IMDs where the two bacteria tested are common colonizers and reduce their biofilm formation.

## 1. Introduction

Implantable medical devices (IMDs), such as prosthetic joints and catheters, have been widely used in the medical field for both diagnosis and therapeutic purposes [1,2]. In recent years, the number of implanted medical devices has increased significantly with the aging population and the growing occurrence of comorbidities [3,4]. Although IMDs are essential for maintaining the life quality of patients, they are associated with serious clinical complications, including the occurrence of infections [5]. Implant-associated infections (IAIs) have an incidence between 2% and 40%, depending on the type of medical device [6], and are responsible for prolonged hospital stays, increased costs, and high morbidity and mortality rates [7]. In the United States, these infections account for 60–70% of all healthcare-associated infections reported annually [4] and represent approximately $3 billion of direct costs [8].

Most IMDs are susceptible to microbial adhesion and, consequently, biofilm formation, which is the leading cause of IAIs. A variety of pathogens can cause device-related infections, depending on the type of implantable device and the anatomical site of implantation [4,9]. Despite IAIs being often caused by *Staphylococcus* spp. [10], many other pathogens may be responsible for these infections, including *Enterococcus* spp. [11], *Escherichia coli* [12], *Pseudomonas aeruginosa* [13], and *Candida* spp. [14]. Once adhered to implant surfaces, microorganisms form biofilms, which protect them from the host immune response and the action of antimicrobial agents, contributing to the persistence and spread of infection [15,16]. Biofilms often harbor viable but nonculturable (VBNC) cells, which are living cells that have lost the ability to divide in media on which they normally grow. These cells have an intact membrane, undamaged genetic information, a lower metabolic rate, and higher physical and chemical resistance when compared to culturable cells [17]. Additionally, it has been shown that some pathogenic VBNC cells are not detected by standard methods and are not eliminated by antibiotic treatment, being able to resuscitate to a normal metabolic state and cause infection [18]. In the past decades, the increasing antimicrobial resistance has limited the efficacy of antibiotic treatment, contributing to biofilm development with consequent device failure and chronic infection [3,19].

The difficulties in treating established biofilms have prompted research on implant surfaces that resist microbial colonization and consequent biofilm formation. The strategies used to prevent microbial adhesion are based on coating IMDs with compounds that generate anti-adhesive/bacteria-repelling (e.g., polymers), contact-killing (e.g., antimicrobial peptides), or antimicrobial-releasing (e.g., metals and biocides) surfaces [3,20]. Despite the efforts to reduce the incidence of IAIs, most of the developed coatings exhibit low biocompatibility and toxicity for human cells [15], and their antimicrobial efficacy has not been completely proven.

Chitosan (CS) is a cationic polysaccharide obtained from chitin [21,22], which is commonly sourced from crustacean shells, mollusks, insects, and fungi [23], and converted into CS through partial deacetylation [24]. In the past years, CS has been introduced in the medical field due to its attractive intrinsic properties, namely high biocompatibility, non-toxicity, biodegradability, low allergenicity, and low cytotoxicity for human cells [25]. Furthermore, CS and its derivatives have a wide spectrum of antimicrobial activity against Gram-positive and Gram-negative bacteria, filamentous fungi, and yeasts, both in planktonic and sessile states [26]. Although the CS mechanism of action is not completely characterized, three main mechanisms were proposed for the inhibition of microbial growth: (i) cell membrane disruption, (ii) complexation with DNA, and (iii) metal chelation. The first mechanism is based on electrostatic interactions between the positively charged CS molecules (due to the amino groups of glucosamine) and negatively charged cell membranes (due to the presence of phospholipids with anionic phosphate groups), which change the permeability of cell membranes with consequent loss of intracellular content and cell death [27,28]. The second mechanism consists of the penetration of CS molecules into microbial cells and binding to DNA with subsequent inhibition of mRNA and protein synthesis [29]. In the third mechanism, CS molecules chelate metal ions (e.g., Ca^2+^ and Mg^2+^), damaging microbial cell walls [27,30].

The antimicrobial activity of CS and its derivatives is dependent on a set of environmental factors, including the pH, microorganism species, and their structural properties such as source, concentration, degree of deacetylation, and molecular weight [27,31,32]. Up to date, several studies have focused on CS efficacy to reduce biofilm formation on indwelling catheters [33,34,35,36,37]. Although results are encouraging, the employment of CS on IMDs remains understudied and the relation between CS chemical properties, in particular molecular weight, and its antibiofilm activity is not fully understood [31]. Hence, the present study aims to functionalize polylactic acid (PLA) surfaces incorporating chitosan with different molecular weights and evaluate their performance to prevent single- and dual-species biofilm formation by *Staphylococcus aureus* and *Pseudomonas aeruginosa* as they are common colonizers of implantable devices [38]. To the best of our knowledge, this is the first study that reveals the potential of CS-based surfaces of different Mw to reduce VBNC cells in multispecies biofilms associated with the medical field.

Polylactic acid (PLA) was the material chosen for this work because it is one of the most commonly used biodegradable polymers in clinical applications, including for the construction of IMDs [39,40]. This is mainly due to its biocompatibility, non-toxicity, and safe degradation products. Furthermore, the mechanical properties of PLA are similar to synthetic polymers and it has the advantage of the higher abundance and lower cost [41]. In the past years, several modifications using natural compounds, peptides, enzymes, metals, chelating agents, and antibiotics, have been introduced into PLA polymeric matrix to provide antimicrobial and antibiofilm properties [42].

## 2. Results

### 2.1. Characterization of Chitosan and Its Derivatives

Endoskeletons of the *Loligo opalescences* squid were processed through pre-optimized enzymatic and alkaline treatments [43] to extract chitosan. From this procedure, a highly purified β-chitosan (β-CS) with 92% of deacetylation and a molecular weight (Mw) of 294 kDa was obtained. Subsequently, the native CS was depolymerized using sodium nitrite and generated three β-chitooligosaccharides with different Mw: CS1 of 186 kDa, CS2 of 129 kDa, and CS3 of 61 kDa (Figure S1 in Supplementary Materials). Chitosan solutions at 0.5% (*w*/*v*) were then immobilized on PLA films by dip-coating.

### 2.2. Characterization of Functionalized Surfaces

Since it is known that surface properties influence the extension of cell adhesion and consequent biofilm formation [44,45], the four CS-coated PLA surfaces and control (PLA) were first analyzed concerning their hydrophobicity through water contact angle measurement using the sessile drop method (Table 1; Figure S2 in Supplementary Materials).

Considering that water contact angle values below 90° indicate that a surface is hydrophilic [46], results demonstrated that both PLA and CS-PLA surfaces have a hydrophilic behavior. However, the immobilization of 0.5% CS (*w*/*v*) solutions on PLA surfaces by dip-coating significantly decreased the PLA water contact angle by 40–50%, which confirms the presence of CS on functionalized surfaces. Moreover, the molecular weight of CS did not influence the wettability of functionalized surfaces since the water contact angle remained at 40°.

**Figure 1:**
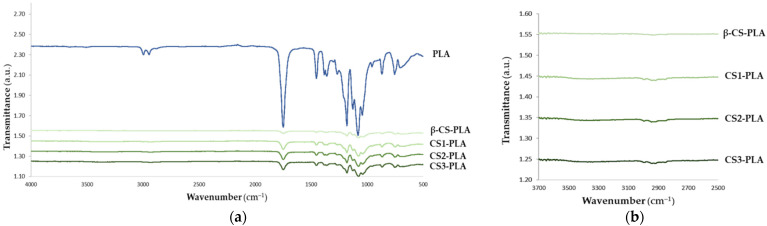
FTIR spectrum of (**a**) PLA and CS-PLA surfaces and (**b**) an inset graphic showing the characteristic bands of CS onto PLA films.

The chemical modifications introduced on PLA surfaces after β-CS, CS1, CS2, and CS3 immobilization were evaluated by Fourier transform infrared spectroscopy (FTIR, Figure 1). From the analysis of the spectrum of the PLA surface, it was possible to observe the characteristic bands of PLA with high-intensity peaks represented at 1750 cm^−1^ corresponding to CO, at 1188–1090 cm^−1^ corresponding to CO, at 1452–1368 cm^−1^ corresponding to COH, and at 3000 cm^−1^ corresponding to CH [47]. The immobilization of chitosan with different molecular weights was also evaluated by FTIR. It was observed that the functional groups of chitosan, such as the characteristic NH stretch band of chitosan with a maximum at 3350 cm^−1^ [48] was identified on the functionalized CS-PLA surfaces. The broad -OH stretching absorption band between 3680 and 2750 cm^−1^ was also observed, as well as one between 2980 and 2750 cm^−1^ assigned to aliphatic C-H stretching [48] which corresponds to typical vibrations of chitosan. These results confirmed the immobilization of CS on PLA surfaces. However, no differences between the spectrum of PLA surfaces functionalized with CS with different Mw were identified (Figure 1b).

XRD spectroscopy was also performed to prove CS immobilization (Figure S3 in Supplementary Materials). Regarding the PLA film, diffraction peaks at 2θ = 16.5°, 20°, and 22° were obtained, indicating a crystalline polymer matrix [49]. The CS immobilization triggered a decrease in the intensity peaks [50], confirming the deposition of chitosan on the PLA film. Moreover, different types of chitosan did not impact the XRD pattern.

The surface characterization of CS-PLA films was complemented by scanning electron microscopy (SEM) analysis. This technique allows the evaluation of the morphology of functionalized surfaces, and the distribution of CS and its derivatives onto PLA films. SEM analysis demonstrated the clear homogeneity of PLA surface and the presence of small aggregates on PLA surfaces coated with CS, which probably correspond to insoluble CS material (Figure S4a–c in Supplementary Materials). However, chitosan originated uniform and continuous coatings on the PLA film, as can be observed in the higher magnification micrographs (Figure S4d,e in Supplementary Materials).

### 2.3. Antibiofilm Activity of Functionalized Surfaces

**Figure 2:**
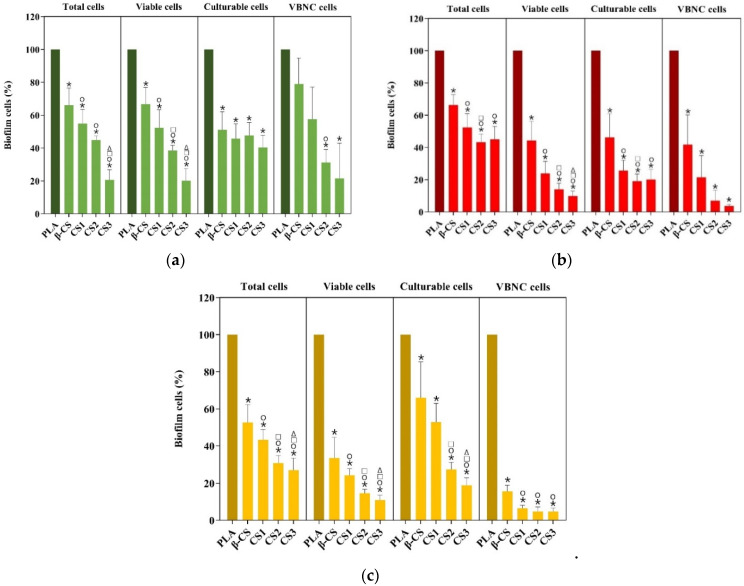
Percentage of total, viable, culturable, and viable but nonculturable (VBNC) cells for (**a**) *S. aureus*, (**b**) *P. aeruginosa,* and (**c**) dual-species biofilms formed during 24 h on PLA and CS-coated PLA surfaces (β-CS, CS1, CS2, and CS3). Differences between functionalized surfaces were evaluated using the nonparametric Mann–Whitney test and represented for *p*-values < 0.05 by *, ○, □, and Δ when compared to PLA, β-CS-PLA, CS1-PLA, and CS2-PLA, respectively.

The antibiofilm performance of PLA, β-CS-PLA, CS1-PLA, CS2-PLA, and CS3-PLA surfaces was evaluated against single- and dual-species biofilms of *S. aureus* and *P. aeruginosa* for 24 h. The cellular composition of biofilms was determined by counting total, viable, culturable, and VBNC cells (Figure 2). Biofilms were detached from surfaces and the number of culturable cells was determined by colony-forming units (CFU) counting, while the number of total and viable cells was determined by epifluorescence microscopy. In turn, the number of VBNC cells was estimated as the difference between the viable cells and culturable ones. Results were presented as the percentage of biofilm cells compared to the control-PLA film.

The analysis of biofilm cells indicated that *S. aureus* biofilms formed on CS-based surfaces exhibited 33 to 79% fewer total cells than PLA (*p <* 0.05), being this reduction higher for CS-PLA surfaces of low molecular weight CS (CS2 of 129 kDa and CS3 of 61 kDa) (Figure 2a). A similar tendency was observed for the number of viable cells, with biofilms formed on CS2- and CS3-PLA surfaces presenting only 39 and 20% cells (*p <* 0.05), respectively. *S. aureus* biofilms formed on CS-PLA surfaces also displayed a lower percentage of culturable cells (40–51%) compared to PLA (*p <* 0.05). In turn, the percentage of VBNC cells was reduced by 21 to 78% on CS-coated PLA surfaces, being this effect more pronounced for CS2- and CS3-PLA (*p <* 0.05).

Regarding *P. aeruginosa* (Figure 2b), biofilms formed on CS-based surfaces displayed a lower percentage of total (45–66%), viable (10–44%), and culturable (20–46%) cells compared to PLA (*p <* 0.05). Once again, the most effective surfaces were those with low molecular weight CS (CS2 and CS3). Moreover, CS2- and CS3-PLA were able to reduce the percentage of VBNC cells by 93 and 96%, respectively.

The analysis of biofilm cell composition also demonstrated that CS-based surfaces exerted a higher antimicrobial activity against *P. aeruginosa* than *S. aureus* as demonstrated by the percentage of viable, culturable, and VBNC cells (Figure 2a,b).

When evaluating the performance of CS-based surfaces against dual-species biofilms (Figure 2c), results showed that these surfaces presented a lower percentage of total (27–53%), viable (11–34%), and culturable (19–66%) cells compared to PLA (*p <* 0.05). In addition, VBNC cells were significantly reduced in CS1-, CS2-, and CS3-PLA surfaces by more than 94%.

The inhibition of dual-species biofilms was also dependent on CS molecular weight. Furthermore, CS-based surfaces yielded a similar antimicrobial effect against *P. aeruginosa* and dual-species biofilms, as confirmed by the percentage of viable, culturable, and VBNC cells. In fact, dual-species biofilms were mostly composed of *P*. *aeruginosa* (more than 3:4, Figure S5a–e in Supplementary Materials), which may justify a more similar behavior between single-species biofilms of *P. aeruginosa* and mixed biofilms.

**Figure 3:**
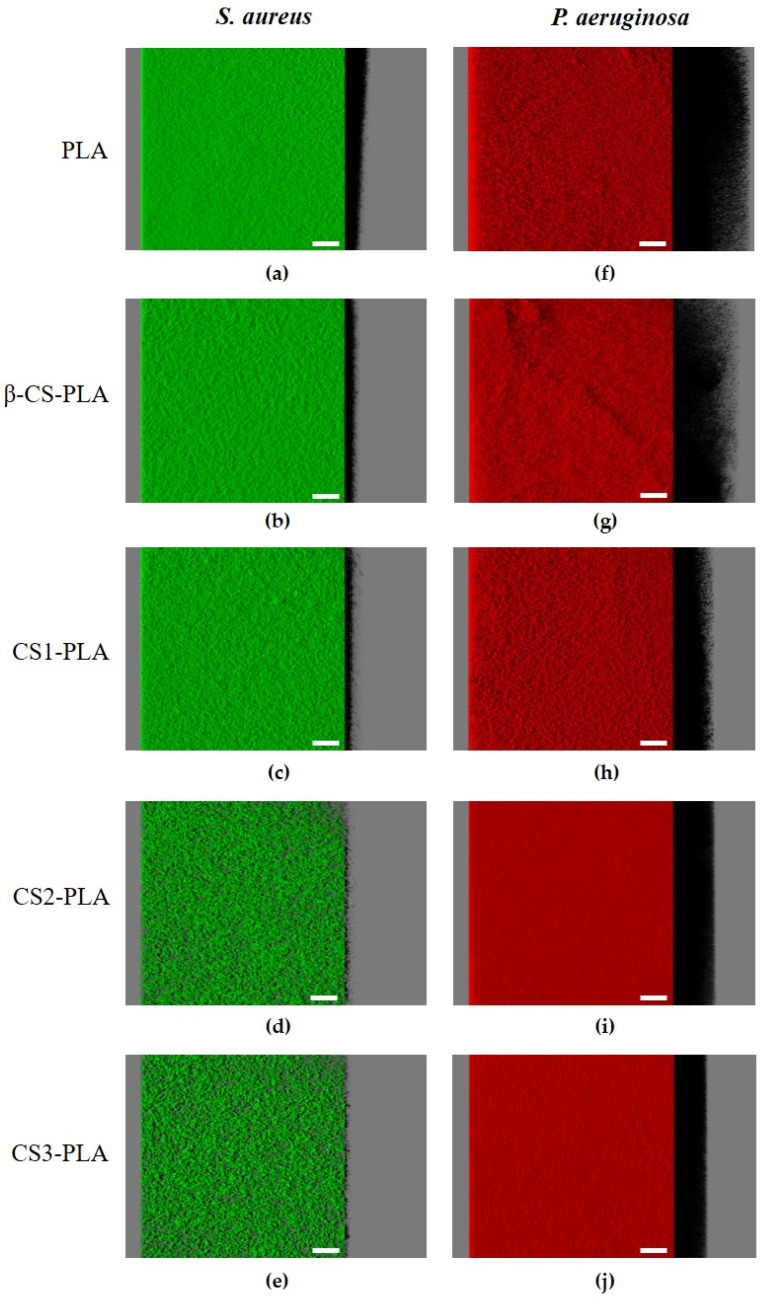
Single-species biofilms of (**a**–**e**) *S. aureus* and (**f**–**j**) *P. aeruginosa* developed on PLA and CS-coated PLA surfaces. These representative images were obtained from confocal *z*-stacks using IMARIS software and present an aerial, three-dimensional (3D) view of the biofilms. The shadow on the right represents the vertical projection of the biofilm. Scale bars are 50 μm.

The antibiofilm activity of CS-based surfaces against single- and dual-species biofilms of *S. aureus* and *P. aeruginosa* was also evaluated by confocal laser scanning microscopy (CLSM). Figure 3 shows the three-dimensional (3D) structure of single-species biofilms of *S. aureus* and *P. aeruginosa* developed on PLA (control surface) and PLA surfaces functionalized with different chitosans (β-CS, CS1, CS2, and CS3). It is possible to visualize that the *P. aeruginosa* strain formed denser and thicker biofilms (shadow projection on the right of micrographs) than *S. aureus*, regardless of the surface material. Additionally, the uncoated PLA film (Figure 3a,f) showed the highest biofilm amount when compared to the surfaces functionalized with CS, regardless of the bacterial strain tested.

**Figure 4:**
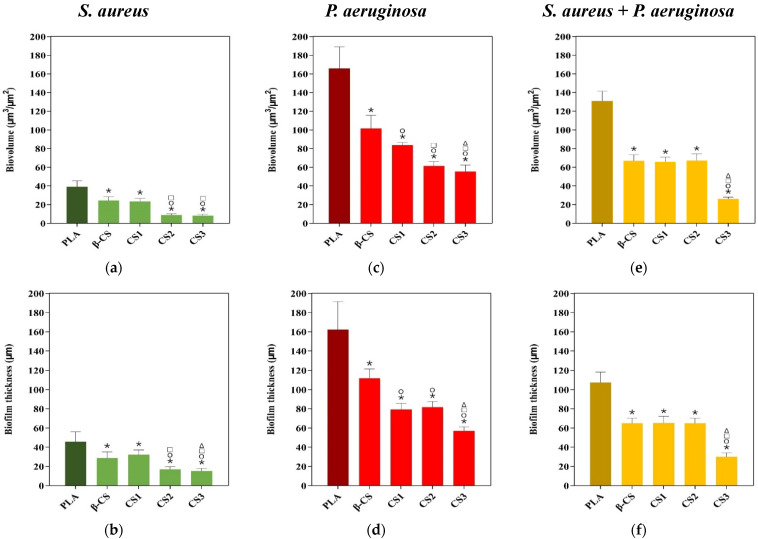
Biovolumes (**a**,**c**,**e**) and thickness (**b**,**d**,**f**) of *S. aureus* and *P. aeruginosa* single-species biofilms, and of dual-species biofilms (*S. aureus* + *P. aeruginosa*) on PLA and CS-coated PLA surfaces. These parameters were obtained from confocal image series using the COMSTAT2 tool associated with the ImageJ software. The means ± standard deviations for three independent experiments are illustrated. Statistical analysis was performed using one-way analysis of variance (ANOVA) and significant differences were represented for *p*-values < 0.05 by *, ○, □, and Δ when compared with PLA, β-CS, CS1, and CS2, respectively.

Looking at the architecture of staphylococcal biofilms (Figure 3a–e), a gradual decrease in biofilm amount and thickness was observed as the CS Mw decreased (from the surface coated with β-CS of Mw = 294 kDa to the surface with CS3 of Mw = 61 kDa). This visual inspection was validated through the biofilm biovolume and thickness values estimated from the confocal image analysis (Figure 4a,b). Indeed, the CS2 and CS3 surfaces containing the two chitosans with lower Mw (129 and 61 kDa, respectively) were capable of reducing the *S. aureus* biovolume and biofilm thickness by on average 78% and 65%, respectively, when compared to the PLA surface (*p <* 0.05, Figure 4a,b).

Concerning the *P. aeruginosa* single-species biofilms, microscopic images revealed that a reduction in biofilm thickness occurred between the PLA and CS-coated PLA surfaces (shadow projection on the right of Figure 3f–j).

Quantitative data (Figure 4c,d) shows that, on average, CS1 and CS2 decreased the biovolume and thickness of *Pseudomonas* biofilms by 56% and 50%, respectively, compared to PLA (*p <* 0.05), while CS3 was the surface with the highest antibiofilm performance, as reported for *S. aureus*, with reductions of around 66% for both biofilm parameters (*p <* 0.05, Figure 4c,d).

**Figure 5:**
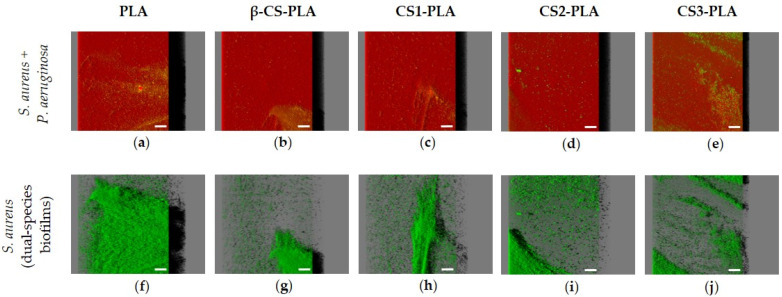
Dual-species biofilms of *S. aureus* and *P. aeruginosa* on PLA and CS-coated PLA surfaces. These representative images were obtained using IMARIS software and show an aerial view of biofilm structures, with the shadow on the right representing the vertical projection of the biofilm. The first row (**a**–**e**) presents the combination of red and green filters (*S. aureus* + *P. aeruginosa*), while the second row (**f**–**j**) corresponds only to the green filter (*S. aureus*). Scale bars are 50 μm.

Figure 5 presents the 3D structure of dual-species biofilms (*S. aureus* + *P. aeruginosa*) formed on the different surfaces in order to elucidate the interactive behavior of the strains when co-cultured for 24 h. The first row presents the simultaneous localization of *P. aeruginosa* (in red) and *S. aureus* (in green) within the dual-species biofilms (Figure 5a–e), whereas the second row corresponds to the spatial distribution of only *S. aureus* cells in the same biofilms (Figure 5f–j). As observed for the single-species biofilms of *P. aeruginosa*, mixed-species biofilms are quite dense and thick on all tested surfaces (shadow projection on the right of Figure 5a–e), and the dominant strain was clearly the Gram-negative bacterium *P. aeruginosa*. A small number of *S. aureus* cells were heterogeneously distributed across surfaces, particularly on CS-based surfaces where the percentage of *S. aureus* population in the biofilm varied between 21% and 28% (Figure S5a–e in Supplementary Materials). When comparing the confocal images of *S. aureus* in single- and dual-species biofilms (Figure 3a–e and Figure 5a–e, respectively), it is noticeable that *S. aureus* colonization was reduced in mixed biofilms. The CLSM study also indicated that the strains were co-located, which means that *P. aeruginosa* and *S. aureus* cells were mixed throughout the biofilm volume (co-aggregation), independently of the tested materials (data not shown).

Measurements of total biovolume and biofilm thickness of dual-species biofilms (Figure 4e,f) showed that the values of these two parameters were overall between the lower values registered for the single-species biofilms of *S. aureus* (Figure 4a,b) and the higher values determined for *P. aeruginosa* biofilms (Figure 4c,d). Furthermore, biofilms developed on CS-based surfaces had on average 57% less biovolume and 48% less thickness than on PLA, with the CS3-PLA showing again to be the most efficient surface in inhibiting biofilm formation.

Overall, the antimicrobial activity increase of CS-based surfaces with decreasing chitosan Mw was demonstrated by both biofilm cell composition and biofilm structure through CLSM analysis.

## 3. Discussion

In this study, CS-based surfaces with different Mw were produced, and their efficacy to inhibit the development of single- and dual-species biofilms of *S. aureus* and *P. aeruginosa* was evaluated through the analysis of biofilm cell composition and structure.

Four CS with decreasing Mw, from 294 to 61 kDa, were successfully obtained through the enzymatic, alkaline, and salt processing of chitin sourced from *Loligo opalescences* squid endoskeletons [43] (obtained as a discard from the fishery industry) and immobilized at 0.5% (*w*/*v*) onto PLA films by dip-coating. Most of the commercially available chitin comes from crustaceans shells, where polymeric chains are in antiparallel fashion (α-chitin), while the opposite occurs in other sources such as squid pen (β-chitin). Inter- and intra-molecular forces are stronger in α- than in β-chitin, resulting in increased solubility and water-absorbing capacity of squid pen β-chitin [51].

Since it is known that surface properties influence microbial adhesion and subsequent biofilm formation [45,52], the PLA and CS-coated PLA surfaces were first analyzed concerning their hydrophobicity, surface chemical interactions, and morphology. Results from water contact angles measurement indicated that the immobilization of CS solutions increased the hydrophilicity of PLA surfaces. Other studies also showed that the immobilization of CS molecules improved surface hydrophilicity by increasing the number of polar groups, making bacterial adhesion less favorable [53,54]. In fact, several authors report that bacteria are more likely to attach to hydrophobic than hydrophilic surfaces [55,56], which supports the notion that functionalized CS-PLA surfaces may reduce biofilm formation on IMDs. In addition, the contact angles of CS-PLA surfaces were not dependent on CS molecular weight and their hydrophilic character was maintained across the different CS-based surfaces. Similar results were obtained by Stoleru et al. [47], which demonstrated that chitosan Mw did not affect the wettability of functionalized PLA surfaces.

Concerning surface chemical modifications, FTIR analysis revealed the presence of CS characteristic bands on the spectrum of PLA [47], indicating that CS molecules were successfully immobilized in these films. Moreover, there were no differences in FTIR spectra among different CS-based surfaces, which suggests that molecular weight did not interfere with interactions between CS and the PLA matrix. This result is supported by a previous study developed by Ang et al. [57], showing that the FTIR spectra of medium and low Mw chitosan displayed a similar pattern.

Lastly, the study of surface morphology by SEM showed that CS-PLA surfaces exhibited a more heterogeneous appearance than the PLA. Indeed, the presence of insoluble CS materials may induce the formation of small aggregates on the surface of functionalized CS-PLA surfaces. As some authors have reported, chitosan has poor solubility [58] and extensive research has been carried out to increase it and extend CS use in a broader range of environmental conditions. Contrary to water contact angles and FTIR results, the CS molecular weight seems to have an impact on surface morphology. In fact, the presence of small aggregates was more notorious for CS-PLA surfaces with high Mw chitosan (β-CS of 294 kDa and CS1 of 186 kDa). According to Peng et al. [59], the functionalization of CS may increase its water solubility, which may explain the higher homogeneity of CS3-PLA surface. Furthermore, other authors have reported that with increasing molecular weight compounds, the reaction between their end-group and the complementary group on the substrate surface becomes less efficient, which may have a significant role in microbial adhesion and cell growth [32,60].

In general, the surface characterization of CS-PLA films revealed that CS was successfully immobilized and that CS molecular weight only impacted its distribution on PLA surfaces.

Besides the surface properties, biofilm development may be influenced by the microorganism type [52]. Thus, the efficacy of CS-coated PLA surfaces to inhibit biofilm formation was assessed against a Gram-positive (*S. aureus*) and a Gram-negative (*P. aeruginosa*) bacteria. The analysis of biofilm cell composition showed that CS-based surfaces significantly reduced the number of total, viable, and culturable *S. aureus* cells by 60 to 80%, revealing their anti-adhesive and antimicrobial properties. The efficacy of CS-coated films to decrease *S. aureus* biofilm formation and reduce its viability was previously reported [53,54] and corroborated by our results. CS-PLA surfaces were able to reduce the percentage of total, viable, and culturable *P. aeruginosa* cells by 55 to 90%. Similar results were previously obtained by Kara et al. [54]. The analysis of biofilm structure revealed the efficacy of CS-PLA surfaces to inhibit *S. aureus* and *P. aeruginosa* biofilm formation compared to PLA films. In fact, uncoated PLA film showed the highest biofilm amount when compared to the surfaces functionalized with CS.

CS-based surfaces were highly effective in reducing the percentage of VBNC cells, particularly for *P. aeruginosa* (>96%). To the best of our knowledge, this is the first study addressing the effect of CS-based surfaces on the reduction of VBNC cells. The VBNC state is an important survival strategy adopted by several bacteria when exposed to unfavorable conditions [61]. Given that VBNC cells are more resistant to antimicrobial therapy and can reinitiate infection when appropriate conditions are established [17,61], our results suggest that CS-PLA surfaces may be beneficial to coat IMDs.

In general, the strong antimicrobial activity of CS-based surfaces against Gram-positive and Gram-negative bacteria possibly results from the interaction between positively charged CS molecules and negatively charged cell membranes, which may prevent mass transfer across the membrane and consequently lead to cell death [62]. In addition, it is also likely that CS penetrates cell membranes and binds to DNA, inhibiting protein synthesis [63]. However, the analysis of biofilm cell composition and structure (e.g., biovolume and biofilm thickness) indicates that CS-coated PLA surfaces exerted higher antimicrobial activity against *P. aeruginosa* than *S. aureus*, which may be explained by bacteria surface polarity. The outer membrane of Gram-negative bacteria is essentially composed of lipopolysaccharides containing phosphate and pyrophosphate groups which increase the negative charge density on the bacterial surfaces, leading to higher attraction to the positive CS surfaces compared to *S. aureus* cells [54]. Furthermore, it was possible to observe that the antimicrobial activity of functionalized surfaces increased with the decrease of CS molecular weight, as demonstrated by biofilm cell composition and structure. In fact, results point to the CS2- and CS3-PLA as the most promising surfaces in reducing the number of biofilm cells, biovolume and biofilm thickness, possibly because low Mw chitosan penetrates the bacterial cell wall more easily due to their reduced size [64].

As several studies have shown that mixed biofilms exhibit enhanced resistance compared to single-species biofilms, the antibiofilm performance of functionalized surfaces was also tested against *S. aureus* and *P. aeruginosa* dual-species biofilms. Data demonstrated that biofilms formed on CS-coated PLA surfaces presented a lower percentage of total, viable and culturable cells than uncoated PLA. These results were supported by biofilm biovolume and thickness analysis. In addition, the presence of VBNC cells was significantly reduced in CS-based surfaces by more than 94%. As with single-species biofilms, the inhibition of mixed biofilms showed the same dependency on CS Mw. Furthermore, CS-based surfaces yielded a similar antimicrobial effect against *P. aeruginosa* and dual-species biofilms. Other authors also demonstrated that CS-based surfaces were effective in inhibiting both single- and dual-species biofilms [65]. The similar behavior of single-species biofilms of *P. aeruginosa* and dual-species biofilms may be related to the strong dominance of *P. aeruginosa* in dual-species biofilms, as demonstrated by cell counting and CLSM analysis. It is possible that *P. aeruginosa* outcompeted *S. aureus* from coculture biofilms [66,67], being the growth of dual-species biofilms representative of the growth rate of the Gram-negative bacteria.

Generally, this work demonstrates the potential of CS of low Mw for coating polymeric devices such as IMDs and hinder single- and dual-species biofilm formation by *S. aureus* and *P. aeruginosa*.

## 4. Materials and Methods

### 4.1. Chitosan and Its Derivatives

Chitosan was isolated from *Loligo opalescens* squid endoskeletons (pens) through a combination of enzymatic and alkaline treatments following a previously optimized protocol [43]. Briefly, squid pens were milled and deproteinized using a protease (Alcalase from Novozymes, Bagsvaerd, Denmark) to produce chitin. Subsequently, chitin was submitted to NaOH treatment for conversion into chitosan. The recovered CS (β-CS) was sequentially depolymerized through the reaction with sodium nitrite according to the protocol developed by Allan et al. [68] and generated three depolymerized samples (CS1, CS2, and CS3) which were freeze-dried and milled to a fine powder.

The molecular weight of native and depolymerized CS was determined by gel permeation chromatography (GPC) [43]. The degree of acetylation of native CS was estimated through nuclear magnetic resonance (NMR) spectroscopy, from the relationship between acetyl integrals (N-acetyl: NAc and AcOH) and the integral sum of H2-H6 protons (glucosamine: GluN and N-acetylglucosamine: GluNAc) [69,70], as shown in Figure S1 (Supplementary Materials). The degree of acetylation was assumed to be virtually the same for native and depolymerized CS as only between 0.1 and 0.3% of the glycosidic bonds were calculated to be cleaved during hydrolysis, based on the initial degree of acetylation and molecular weight estimations.

### 4.2. Surface Functionalization

Solutions of β-CS and its three derivatives (CS1, CS2, and CS3) at 0.5% (*w*/*v*) were immobilized onto polylactic acid (PLA) films (Figure S6 in Supplementary Materials). Transparent PLA films with 0.05 mm of thickness (Goodfellow, UK) were chosen as a substrate because this polymer is commonly used for the construction of IMDs [39]. Table S1 (Supplementary Materials) reports the mechanical, physical, and thermal properties of PLA films.

First, PLA films (1 × 1 cm) were submitted to plasma oxygen treatment (Harrick Plasma, PJS-14-0240) at moderate intensity for 15 min [71] to improve the adhesion of CS molecules. Subsequently, films were coated with the different CS solutions by dip-coating for 15 min and dried with nitrogen for 5 min [71].

### 4.3. Surface Characterization

#### 4.3.1. Water Contact Angle Measurement

The surface hydrophobicity was determined through the measurement of water contact angles by the sessile drop technique in a drop shape analyzer (DSA 100E, Kruss Gmbh, Hamburg, Germany). A water droplet (2 µL) was deposited in different locations of the surface and drop images were acquired using a camera connected to the analyzer. Water contact angles were estimated through the circle-fitting method [72]. For each surface, at least ten measurements were performed [73].

#### 4.3.2. Fourier Transform Infrared Spectroscopy (FTIR)

FTIR spectra of the functionalized CS-PLA surfaces were evaluated with VERTEX 80v FTIR spectrometer (Bruker, Rheinstetten, Germany) in the wavelength range 4000–400 cm^−1^ at a resolution of 4 cm^−1^ and normalized between 0 and 1, using platinum attenuated total reflection mode (ATR) (Bruker, Rheinstetten, Germany) [74].

#### 4.3.3. X-ray Diffraction (XRD)

XRD was used to assess the influence of CS on the crystalline structure of the PLA matrix. The XRD patterns of PLA films with and without chitosan were obtained through a diffractometer (PanAnalytical X Pert PRO MRD system, Malvern, UK). The scanning range varied from 2θ = 10° to 50° [75].

#### 4.3.4. Scanning Electron Microscopy (SEM)

The surface topology and the distribution of CS and its derivatives on PLA films were assessed by SEM (Quanta 650 FEG, FEI Company, Hillsboro, OR, USA) with an accelerating voltage of +5 kV at 500× and 2500×. Samples were cut, placed on sample holders with double-sided adhesive, and sputtered with a 10 nm layer of gold [74].

### 4.4. Bacteria and Culture Conditions

The antibiofilm activity of the functionalized surfaces was evaluated using a *Staphylococcus aureus* reference strain (ATCC 25923) and a mCherry-*P. aeruginosa* PAO1 strain [76], since these microorganisms are commonly isolated from IAIs [38]. The selection of a mCherry-expressing strain allows the *P. aeruginosa* identification in mixed biofilms.

Bacterial strains preserved at −80 °C in Luria-Bertani broth (LB, Thermo Fisher Scientific, Waltham, MA, USA) containing 20% (*v*/*v*) glycerol were spread on plate count agar (PCA, Merck KGaA, Darmstadt, Germany) plates and incubated for 24 h at 37 °C. Single colonies were collected from agar plates, inoculated in 250 mL of LB broth and incubated at 37 °C, 160 rpm for 16 ± 2 h. Tetracycline at 1.25 mg.L^−1^ final concentration was used to select mCherry-*P. aeruginosa* colonies [76].

The overnight cultures were centrifuged (Eppendorf Centrifuge 5810R, Eppendorf, Hamburg, Germany) at 18 °C, 3772 g for 10 min, and resuspended in fresh LB medium in order to obtain a final suspension with an optical density at 610 nm of 0.1, which corresponds to approximately 1 × 10^8^ CFU.mL^−1^. Bacterial suspensions of *S. aureus* and *P. aeruginosa* were directly used to form single-species biofilms, while in the case of dual-species biofilms, they were mixed in a 1:1 ratio.

### 4.5. Biofilms Assays

#### 4.5.1. Biofilm Formation

Biofilms were formed in 12-well microtiter plates (VWR International, Carnaxide, Portugal) under static conditions. UV-sterilized surfaces, including the PLA (positive control) and the four functionalized CS surfaces, were fixed on the microplate wells using double-sided adhesive tape and inoculated with 3 mL of bacterial suspension. In addition, 3 mL of LB medium was added to the sterilized surfaces to control their sterility through the experiments. Microplates were incubated at 37 °C for 24 h.

Biofilm formation experiments were performed in three independent assays, each one with three technical replicates.

#### 4.5.2. Biofilm Quantification

After 24 h of biofilm formation, PLA and CS-based surfaces were detached from microplate wells, dipped in 2 mL of 8.5 g·L^−1^ NaCl solution and vortexed for 3 min at maximum power (ZX4, Velp Scientifica) to obtain biofilm cell suspensions. Then, bacterial suspensions were properly diluted and spread on PCA, and incubated overnight at 37 °C. The culturability of biofilm cells was determined by CFU counting (CFU·cm^−2^). In turn, the viability of biofilm cells was evaluated by staining the biofilm suspension with the Live/Dead^®^ BacLight™ Bacterial Viability kit (Invitrogen Life Technologies, Alfagene, Portugal) as previously described [77], and analyzing in an epifluorescence microscope (Leica DM LB2, Germany). This staining comprises two fluorescent dyes, the Syto^®^9 that penetrates all cells and the propidium iodide that penetrates only cells with impaired membranes. A minimum of fifteen fields of view was analyzed using the ImageJ software (version 1.52p, National Institutes of Health, EUA) and the number of total and viable cells was quantified (cells·cm^−2^). In addition, the number of VBNC cells was also determined by the difference between the number of viable and culturable cells [17].

The total, viable, culturable, and VBNCs cells were presented as the percentage of biofilm cells compared to the control (PLA film).

#### 4.5.3. Confocal Laser Scanning Microscopy (CLSM)

To assess the biofilm spatial organization on CS-PLA surfaces, single- and dual-species biofilms of *S. aureus* and *P. aeruginosa* were observed by CSLM [78,79]. First, 24 h biofilms of *S. aureus* and *S. aureus* + *P. aeruginosa* formed on PLA and functionalized surfaces were counterstained with 6 µM SYTO^®^9 (Thermo Fisher Scientific, USA). *S. aureus*, *P. aeruginosa* and *S. aureus* + *P. aeruginosa* biofilms were then observed using a 40× water immersion objective lens (Leica Microsystems, Germany) in an inverted microscope Leica DMI6000-CS with 488 nm argon and 633 nm helium-neon lasers. The emitted fluorescence was recorded within the ranges of 500 to 580 nm and 640 to 730 nm to collect the SYTO^®^9 and mCherry emission fluorescence, respectively. A minimum of five stacks of horizontal plane images (512 × 512 pixels, corresponding to 387.5 µm × 387.5 µm) with a *z*-step of 1 µm was acquired for each biofilm sample.

Three-dimensional (3D) projections of biofilm structures were reconstructed from the CLSM acquisitions using the blend mode of the “Easy 3D” function of IMARIS 9.1 software (Bitplane, Zurich, Switzerland). The plug-in COMSTAT2 associated with the ImageJ software was used to determine the biovolume (µm^3^·µm^−2^) and biofilm thickness (µm) [80].

### 4.6. Statistical Analysis

Descriptive statistics were used to calculate the mean and standard or error deviation (SD) for the number of total, viable, culturable and VBNC cells, and biovolume and biofilm thickness. Differences between the number of cells obtained for PLA and CS-PLA (β-CS, CS1, CS2, and CS3) surfaces were evaluated using the nonparametric Mann-Whitney test, according to the normality of variables’ distribution. In turn, quantitative parameters obtained from confocal microscopy (biovolume and biofilm thickness) were compared using a one-way analysis of variance (ANOVA).

All tests were performed with a confidence level of 95% (*p*-value < 0.05). Data analysis was performed using the IBM SPSS Statistics version 24.0 for Windows (IBM SPSS, Inc., Chicago, IL, USA).

## 5. Conclusions

In this study, the high antimicrobial and antibiofilm activities of CS-based surfaces were clearly demonstrated against single- and dual-species biofilms of *S. aureus* ATCC 25923 and *P. aeruginosa* PAO1. Although the molecular weight of chitosan did not significantly influence the surface properties of functionalized CS-PLA films, surfaces with low Mw chitosan were more effective in reducing biofilm formation, as demonstrated by both biofilm cell composition and structure. The antimicrobial properties of CS were already described, but the potential of the developed CS-based surfaces to reduce VBNC cells described in our work demonstrates their potential use in clinical applications such as the development of coatings for IMDs.

## Figures and Tables

**Table 1:** Water contact angle values of polylactic acid (PLA) and chitosan (CS)-coated PLA surfaces.

Surfaces	Water Contact Angle Values (mean ± SD)
PLA	69.53 ± 1.2
β-CS-PLA	37.01 ± 1.1
CS1-PLA	38.01 ± 5.3
CS2-PLA	38.93 ± 2.7
CS3-PLA	40.55 ± 2.8

## Data Availability

The data presented in this study are available on request from the corresponding author. The data are not publicly available yet as some data sets are being used for additional publications.

## Supplementary Materials

The following are available online. Figure S1. 1H NMR spectrum (a) and gel permeation eluogram (b) of native chitosan from the pen of *Loligo opalescens* squid (weight average molecular weight (Mw) 294 kDa; polydispersity index (PDI) 1.428); eluograms of depolymerized chitosan: CS1, Mw 186 kDa, PDI 1.349 (c); CS2, Mw 129 kDa, PDI 1.534 (d); CS3, Mw 61 kDa, PDI 1.669 (e). Figure S2. Representative images of water droplets and corresponding contact angles on (a) PLA and CS-coated PLA surfaces: (b) β-CS-PLA; (c) CS1-PLA; (d) CS2-PLA; and (e) CS3-PLA. The results shown in Table 1 resulted from the average of the angles of several drops of water released onto each of the tested surfaces. Figure S3. X-ray diffraction (XRD) patterns of different types of chitosan immobilized onto PLA surface (CS3 (1), CS2 (2), CS1 (3), and β-CS (4)) and of PLA film (5). Figure S4. Scanning electron microscopy images of PLA (a) and PLA films coated with the highest (b, d, β-CS) and lowest (c, e, CS3) molecular weight chitosan at a magnification of 500× (a–c) and 2500× (d–e). The red arrows point to small aggregates visible on CS-PLA surfaces. Figure S5. Proportion of *S. aureus* (in green) and *P. aeruginosa* (in red) (a–e) culturable cells and (f–j) biovolume in dual-species biofilms formed on PLA and CS-PLA surfaces. Figure S6. Representative scheme of the functionalization of PLA surfaces with different molecular weights chitosan (β-CS, CS1, CS2, and CS3). Table S1. Properties of PLA films from the supplier report.
