# Supplementary material for: Development of Chitosan-Based Surfaces to Prevent Single- and Dual-Species Biofilms of Staphylococcus aureus and Pseudomonas aeruginosa
Source: Molecules. 2021 Jul 20;26(14):4378. doi: 10.3390/molecules26144378 (PMC8306285; doi:10.3390/molecules26144378)
Supplement: Supplementary file 1 [file molecules-26-04378-s001.zip › molecules-1263334-supplementary.pdf]

Supplementary material

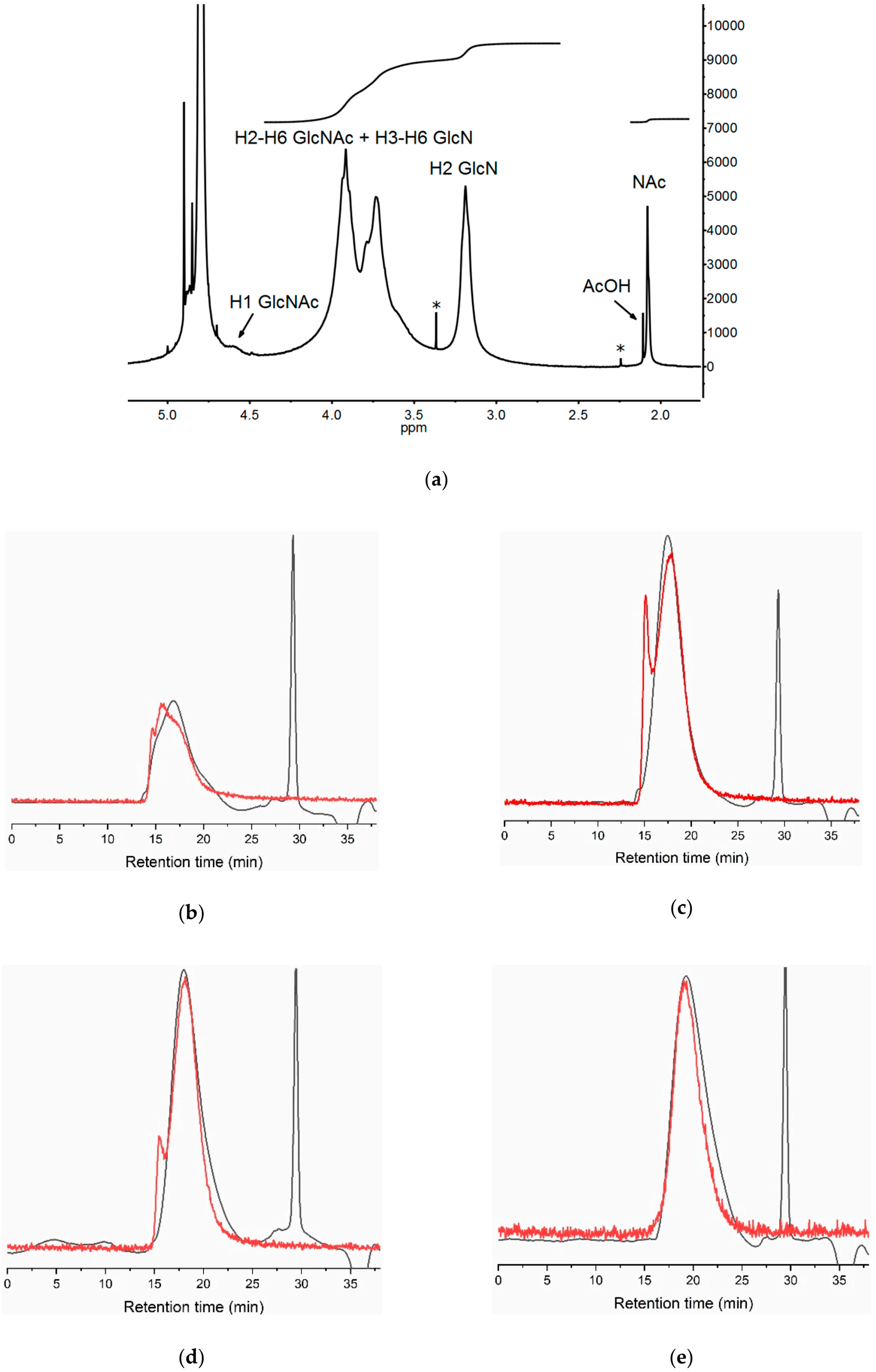

Figure S1. ^{1}H NMR spectrum (**a**) and gel permeation eluogram (**b**) of native chitosan from the pen of *Loligo opalescens* squid (weight average molecular weight (Mw) 294 kDa; polydispersity index (PDI) 1.428); eluograms of depolymerized chitosan: CS1, Mw 186 kDa, PDI 1.349 (**c**); CS2, Mw 129 kDa, PDI 1.534 (**d**); CS3, Mw 61 kDa, PDI 1.669 (**e**).

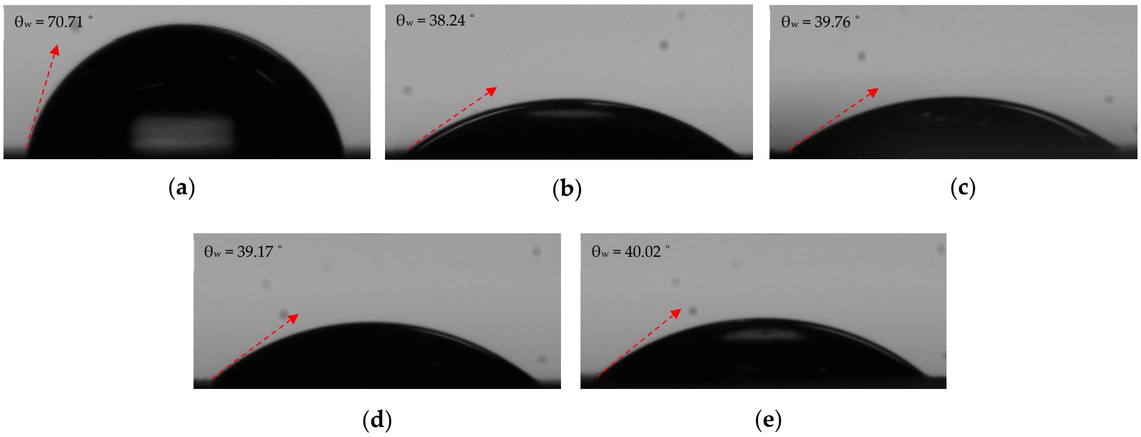

Figure S2. Representative images of water droplets and corresponding contact angles on (**a**) PLA and CS-coated PLA surfaces: (**b**) β-CS-PLA; (**c**) CS1-PLA; (**d**) CS2-PLA; and (**e**) CS3-PLA. The results shown in Table 1 resulted from the average of the angles of several drops of water released onto each of the tested surfaces.

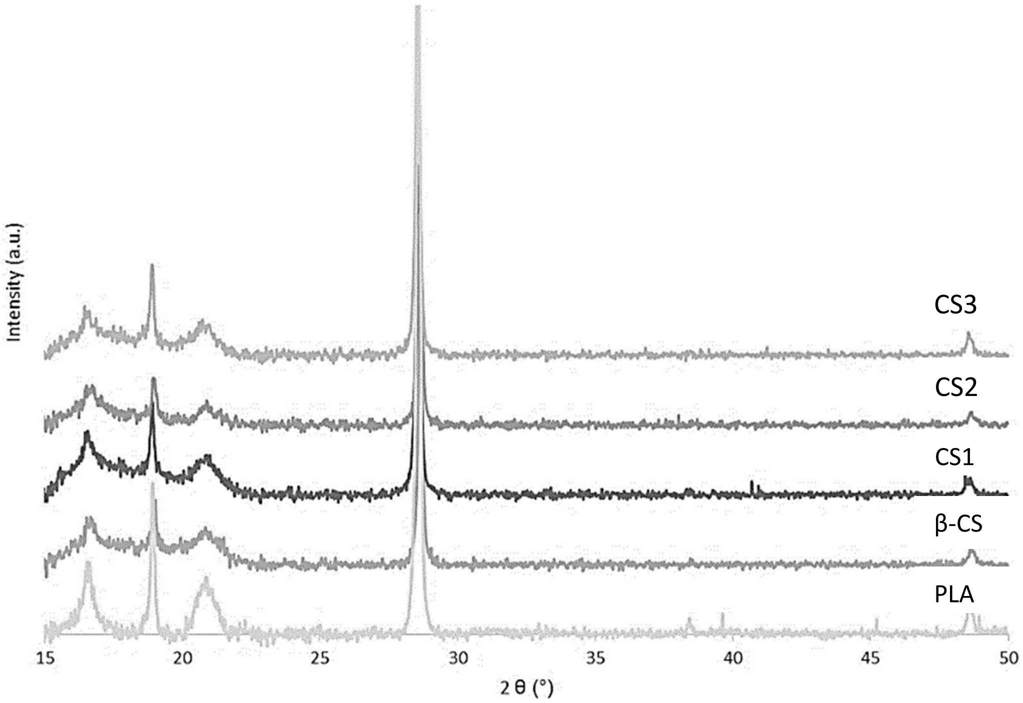

Figure S3. X-ray diffraction (XRD) patterns of different types of chitosan immobilized onto PLA surface (CS3 (1), CS2 (2), CS1 (3), and β-CS (4)) and of PLA film (5).

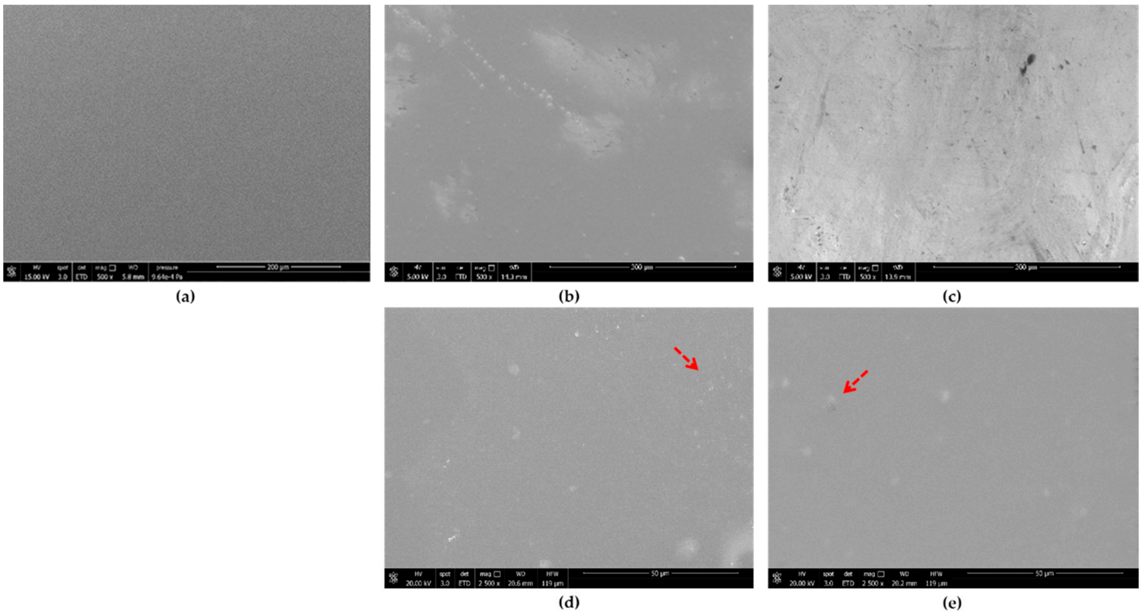

Figure S4. Scanning electron microscopy images of PLA (**a**) and PLA films coated with the highest (**b, d,** β-CS) and lowest (**c, e,** CS3) molecular weight chitosan at a magnification of 500× (**a–c**) and 2500× (**d,e**). The red arrows point to small aggregates visible on CS-PLA surfaces.

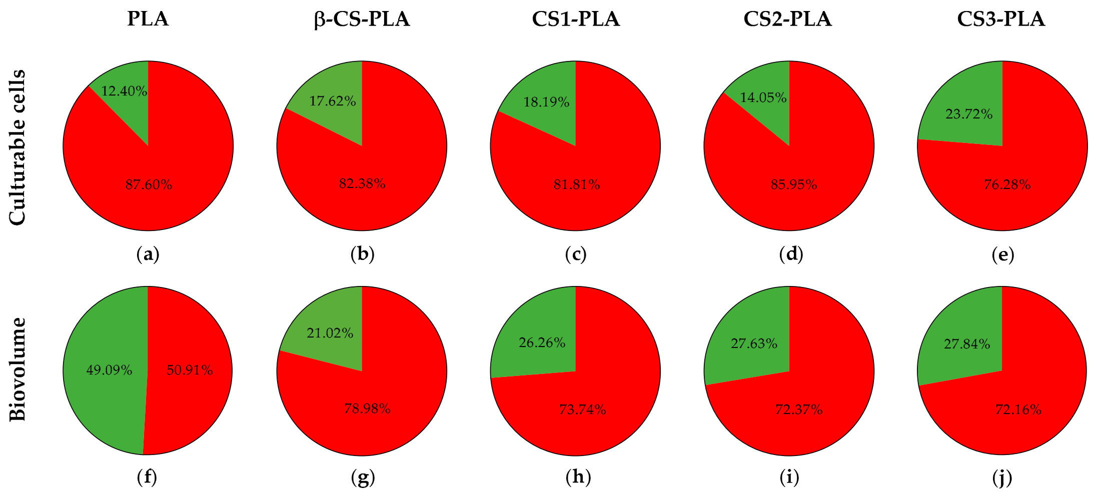

Figure S5. Proportion of *S. aureus* (in green) and *P. aeruginosa* (in red) (**a**–**e**) culturable cells and (**f**–**j**) biovolume in dual-species biofilms formed on PLA and CS-PLA surfaces.

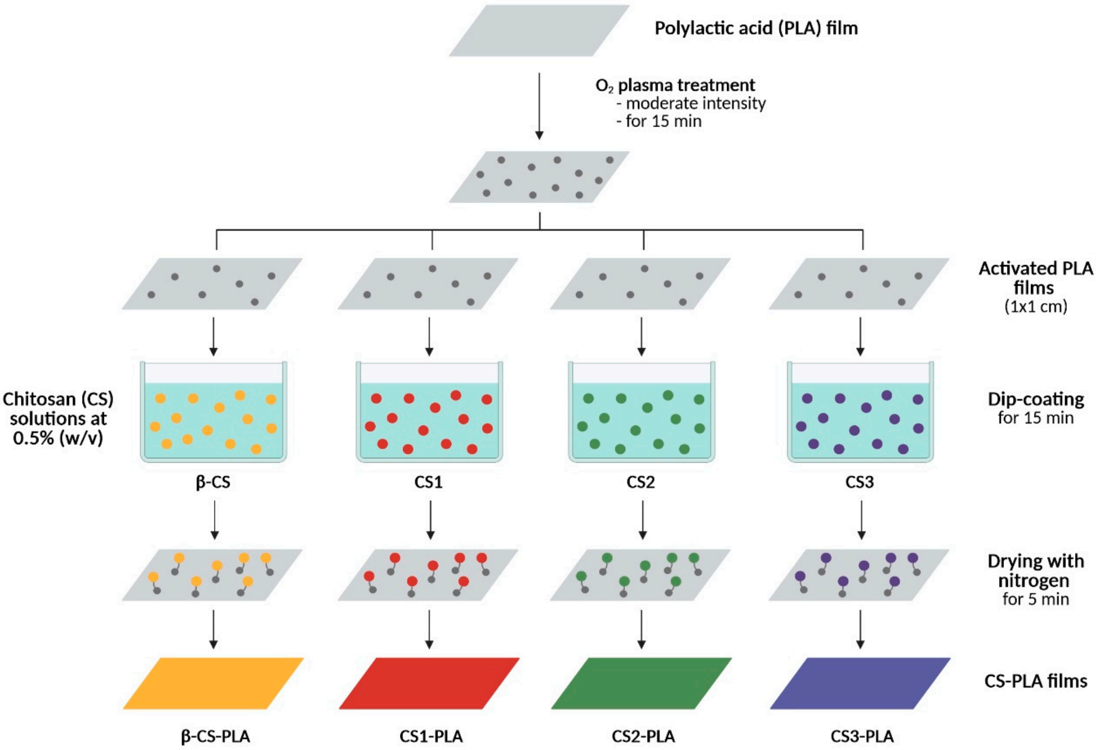

Figure S6. Representative scheme of the functionalization of PLA surfaces with different molecular weights chitosan (β-CS, CS1, CS2, and CS3).

Table S1. Properties of PLA films from the supplier report.

Elongation at break (%)	6
Tensile modulus (GPa)	3
Tensile strength (MPa)	53
Density (g.cm^{-3})	1.24
Thermal properties -1.8 MPa (°C)	55
